# Dynamic airborne mycobiome in the metropolitan city transit system is driven by seasonality and station type

**DOI:** 10.1128/spectrum.01626-25

**Published:** 2025-09-25

**Authors:** Xin Zhou, Da Li, Xiao-Wei Lu, Clement K. M. Tsui, Supawadee Ingsriswang, Jun-Min Liang, Lei Cai

**Affiliations:** 1 State Key Laboratory of Microbial Diversity and Innovative Utilization, Institute of Microbiology, Chinese Academy of Sciences 85387, Beijing, P. R. China; 2 College of Life Science, University of Chinese Academy of Sciences 617066, Beijing, P. R. China; 3 Infectious Disease Research Laboratory, National Center for Infectious Diseases, Tan Tock Seng Hospital 63703, Singapore, Singapore; 4 Faculty of Medicine, University of British Columbia 12358 , Vancouver, British Columbia, Canada; 5 LKC School of Medicine, Nanyang Technological University 54761, Singapore, Singapore; 6 Thailand Bioresource Research Center (TBRC), National Center for Genetic Engineering and Biotechnology, National Science and Technology Development Agency, Pathum Thani, Thailand; University of Mississippi, University, Mississippi, USA

**Keywords:** subway station, mycobiome diversity, urban environment, human pathogen, public health

## Abstract

Subway transit systems serve as the primary transportation mode in metropolitan areas. The quality of the airway in transit plays a crucial role in human health as commuters and workers are exposed to microbes transmitted from passengers and circulated within built environments. The diversity and dynamics of bacterial microbiome in these environments have been relatively well studied. However, the fungal communities remain poorly investigated. In this study, we conducted a year-long comprehensive investigation of the mycobiomes within the world’s second largest subway system and analyzed the seasonal dynamics of fungal composition across intercity hub stations, urban hub stations, and suburban stations. We found a high diversity of the subway mycobiome that varied seasonally and was influenced by various environmental factors, such as particulate matter (PM2.5) levels and average humidity. Fungal diversity was higher in months with elevated PM2.5 pollution. Autumn exhibited increased diversity and peaks in the distribution of human pathogenic fungi. Furthermore, it was determined that station types exert a significant influence on the diversity of pathogenic fungi, with interchange stations (train and airport transfer stations) showing the highest diversity, while suburban stations showed the lowest. The core taxa of the mycobiome comprised several genera including ubiquitous fungi commonly found in soil and outdoor environments (e.g., *Alternaria* and *Cladosporium*), as well as potential plant and human pathogens (e.g., *Phoma* and *Fusarium*), indicating a potential risk to public health. Our study demonstrated the seasonal and spatial dynamics of mycobiomes in the Beijing subway system and revealed the factors/mechanisms that shape the indoor fungal communities. Understanding the patterns and processes of mycobiome community is important for infection prevention and public health management.

**IMPORTANCE:**

Respiratory infections and allergic reactions caused by airborne fungi have received considerable public attention; however, fungal communities remain poorly investigated. This research performed the first year-long investigation of airborne mycobiome in the world's largest subway system. We found that the fungal diversity peaks in autumn and at stations with higher PM2.5 levels. Intercity hubs exhibit the highest diversity of pathogenic fungi and the least seasonal fluctuation. Suburban stations revealed a reduced diversity of human pathogens but an elevated presence of plant pathogens. Core fungal taxa in subways include both common soil fungi (e.g., *Alternaria* and *Cladosporium*) and potential plant and human pathogens (e.g., *Phoma*, *Fusarium*, and *Rhinocladiella*) that pose potential health risks. These results are crucial for infection prevention and public health management in city transit systems.

## INTRODUCTION

The subway system is a crucial component of urban public transportation. With millions of daily passengers, it is significantly influencing people’s lives and health (1). Subway stations, characterized by enclosed spaces, high population density, and stable temperature and humidity, serve as ideal environments as reservoirs for microbial proliferation. Also, passengers of diverse regions and backgrounds may shed their microbes and commensals into the environment via respiratory activities, physical contact, and interaction in the transit, thus establishing a unique microbiome in the subway environment (2, 3). Microbial exposure would impact the health of passengers and workers and staff in the transit system. The distribution and transmission of these microbes, especially pathogens, are dynamic and complex and of significant public health concern (4). Therefore, it is important to characterize and monitor the microbiological composition and their dynamics in the subway systems, of which the data can contribute to the biosurveillance of microbial pathogens in the environment.

Recent technological advancements in metagenomics and metabarcoding have significantly improved our ability to explore the microbial diversity, composition, dynamics, and potential functions within the subway environments. An international consortium, MetaSUB, has collected samples from urban transport systems, including subways and buses in 60 cities worldwide to investigate the density, types, and dynamics of urban metagenomes (3). Their findings revealed an extensive array of microbial diversity, encompassing thousands of known microorganisms with many previously unknown viral and bacterial species (5). Similarly, bacterial 16S rRNA gene amplicon profiling has demonstrated variations in communities across cities and subway lines, which have been influenced by factors such as geography, seasonal dynamics, and architectural layouts (6, 7). For instance, *Paracoccus* spp. had higher abundance in the air microbiomes in Hong Kong, China, and United Kingdom subways, but they were relatively low in Norway and Sweden subway systems (8). The bacterial diversity appeared to peak in the spring and summer months; air samples showed higher levels of within-sample diversity (9). Not only did clinical bacterial communities have higher relative abundance in the cross-border transit line that connects Hong Kong and Shenzhen than other transit lines, but also antibiotic resistance genes demonstrated a significant increase among the systems (10). In addition to environmental factors, the composition of microbes in indoor environments is primarily influenced by human activities (11). Some of the microbes could be released through activities such as human conversation, coughing, and shedding of skin cells (4, 12). For example, the airborne microorganisms in the New York subway consist of a mixture of soil, environmental water, and human-associated bacteria. Also, bacteria and fungi dominate the rRNA microbiomes in subways, of which *Aspergillus*, *Malassezia*, *Nigrospora*, *Staphylococcus epidermidis*, *Staphylococcus hominis*, and *Staphylococcus cohnii* are all typical human pathogens (13, 14). Viral detections have identified *Propionibacterium* phages and Merkel cell polyomavirus (15). Collectively, previous research findings underscore the critical role of humans in the dissemination of subway airborne microorganisms. These results have established a foundation in understanding the dynamics and composition of bacterial communities in subway systems and provided insights for the transmission and circulation of pathogens in the community.

Airborne fungi play a direct and important role in human health, particularly in the respiratory systems (16). The World Health Organization published a list of fungal priority pathogens to guide public health actions in 2022, highlighting 19 fungal species of significant health risks. Among the list, several fungi, such as *Aspergillus fumigatus*, are primarily airborne and can be encountered in indoor environments (17). Also, airborne fungal spores from plant pathogens and soil-borne fungi, such as *Alternaria*, *Aspergillus*, *Ganoderma*, *Malassezia*, *Mucor*, and *Rhizopus*, can cause a variety of allergic reactions (18). Despite their significant impact on the air environment and human health, fungi have been largely overlooked in the subway air microbiome studies (19). Several studies have analyzed airborne fungi in subway stations using culture-dependent techniques (16, 20–22); however, these studies have focused more on the concentration of fungal spores rather than the fungal diversity and composition. Since the number of fungal genera reported (from the indoor environment) has been limited and not standardized (23), it is important to characterize the diversity, composition, and seasonal dynamics of the subway mycobiomes and to widen our understanding of the indoor fungi communities in transit systems, which can then, in turn, improve public health surveillance strategies and infection control and prevention practices (24).

In this study, we conducted a year-long investigation on the fungal communities present in the Beijing subway system. The Beijing subway network, which spans 879 km and includes 27 lines and 522 stations, is among the largest in the world (https://www.bjsubway.com/). We chose 15 representative stations across 14 lines, including intercity hubs, urban hubs, and suburban locations for sampling, aiming to provide a comprehensive view of mycobiome diversity and the potential transmission of medically important pathogens within the Beijing subway network. Our main hypotheses were that (i) the fungal communities in subway stations could vary with locations, regions, and seasons and (ii) specific microbes can be associated with specific subway lines and systems. By examining the changes and relative occurrence in fungal communities in the Beijing subway system, the findings obtained from this study will offer a valuable framework for improving public health monitoring strategies and bio-surveillance in subway management.

## MATERIALS AND METHODS

### Sample collection

**Fig 1:**
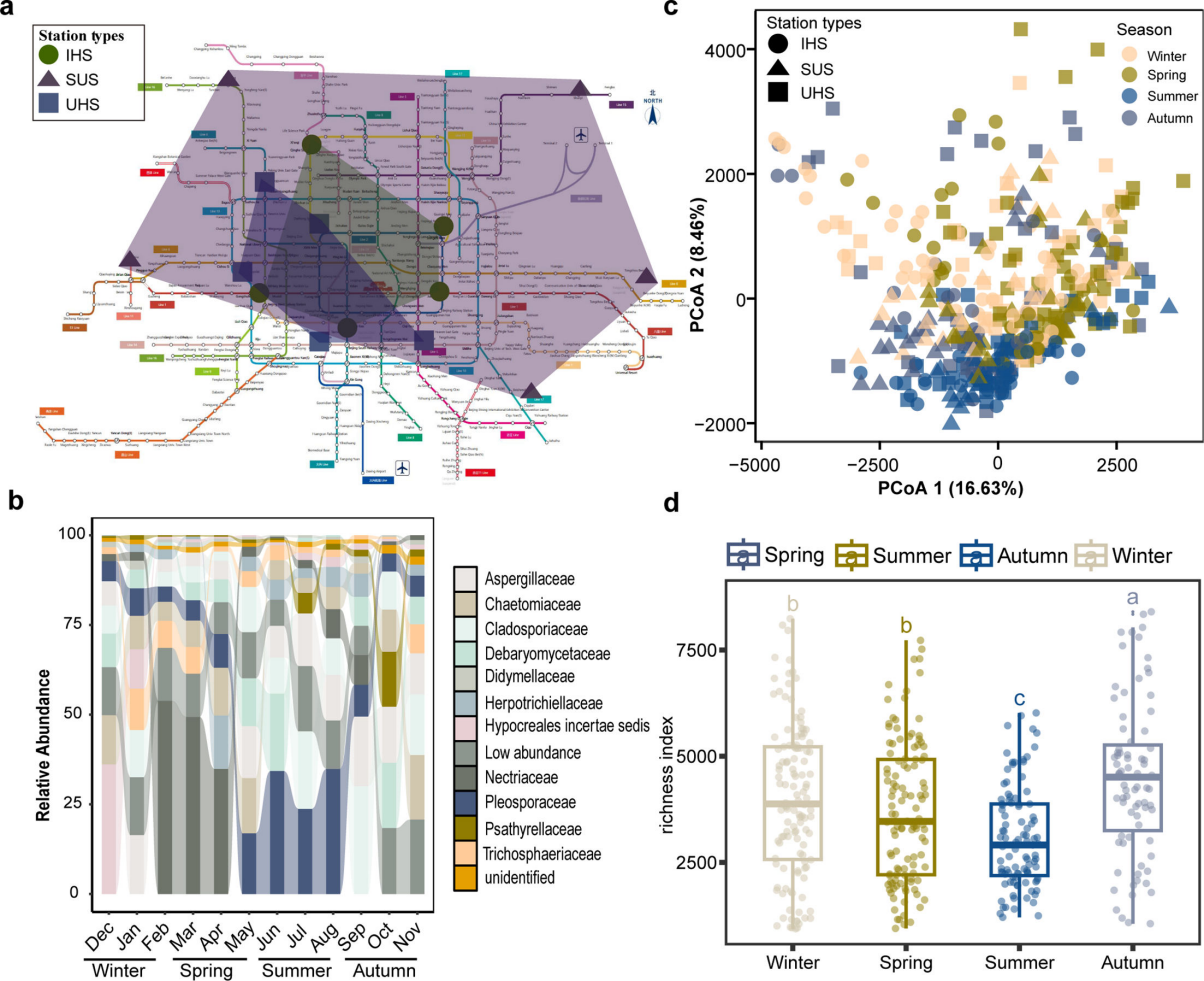
(**a**) Beijing subway line map with 15 different stations and three different station types marked in the map. IHS, intercity hub stations (green circles); SUS, suburban stations (purple triangles); UHS, urban hub stations (blue squares). (**b**) Relative abundances of the most abundant fungal family linked to Beijing subway stations. (**c**) Principal component analysis plotted with Manhattan distance showing the fungal communities associated with subway stations from three station types and different seasons (marked with different colors). PCoA, principal coordinate analysis. (**d**) Fungal richness index of subway stations categorized by different seasons.

Air samples were collected monthly from October 2021 to September 2022 from the air filters of the ventilation systems at 15 stations (Fig. 1a). For each biological sample, five sterile swabs (MUNKCARE, Changzhou Munk Foam Technology Co., Ltd) were used to swab the surface of fan baffles, measuring approximately 15 × 60 cm². There were three replicates per station (Fig. 1b). After sample collection, the fan baffle surfaces were sterilized with 75% ethanol to ensure that microbial attachment was collected consistently throughout the month. Routine sterilization in each station was performed by cleaning staff at the end of the subsequent month. The 15 stations were categorized into three types: intercity hub stations (137 samples from intercity hub stations [IHS]), which included four railway stations and one transfer station to an airport; urban hub stations (UHS) (171 samples from UHS), comprising five transfer stations for two to three lines; and suburban stations (SUS) (110 samples from SUS), consisting of five non-transfer stations located in remote residential areas. Detailed information, such as station names, types, collection times, and related environmental factors, is available in Table S1. Additionally, relevant climatic data were obtained from the China Meteorological Data Service Center website (https://data.cma.cn/).

### Macro-DNA extraction, amplification, and sequencing

Before DNA extraction, five swabs per sample were vortexed together at 150 rpm for 30 minutes in phosphate-buffered saline buffer containing 1% Triton X-100. Subsequently, the samples underwent disinfection using a 40 kHz ultrasonic cleaner (Kunshan KQ-500DE) for 15 minutes. Genomic DNA was extracted using the Fast DNA Spin Kit for soil (MP Biomedicals, USA), following the manufacturer’s protocol. The concentration of the extracted DNA was measured with a NanoDrop ND-2000 spectrophotometer (Thermo Fisher Scientific, Massachusetts, USA) and further purified using the GeneJet Gel Extraction Kit. For the PCR amplification of the fungal ITS2 region, the primer pair 5.8S-Fun (5′-AACTTTYRRCAAYGGATCWCT-3′) and ITS4-Fun (5′-AGCCTCCGCTTATTGATATGCTTAART-3′) was employed, as described previously (25). The reaction mixture consisted of 20 ng genomic DNA, 0.8 µM primers, and 12.5 µL of 2 × Phanta Max Master Mix (P515-03, Vazyme Biotech), with a total volume of 25 µL. The thermocycling conditions included an initial denaturation at 95°C for 5 minutes, followed by 25 cycles of 95°C for 30 seconds, 55°C for 30 seconds, and 72°C for 30 seconds, with a final extension at 72°C for 5 minutes. A negative control, without template DNA, was included, and only batches without detectable amplification in the negative control were selected for further sequencing. The PCR products were purified using the AxyPrep DNA Gel Extraction Kit (Axygen Biotech), and the resulting libraries were sequenced (500 bp) on the Illumina sequencing platform at Magigene Corporation (Guangzhou, China).

### Data processing, taxonomy annotations, and construction of core mycobiome tree

Raw sequencing data were quality controlled and filtered using the fastq-filter algorithm, ensuring a minimum accuracy of 99% (26). Clean paired-end reads were then merged using the fastq_mergepairs algorithm in USEARCH v.11. Chimeric reads were identified and removed using the UCHIME algorithm, with reference to the UNITE database (27). Non-chimeric reads were then clustered into zero-radius operational taxonomic units (zOTUs) using a 99% similarity threshold, and taxonomic assignments were made using the SINTAX algorithm (28). Prior to statistical analysis, zOTUs with fewer than eight sequences and those lacking known fungal taxonomic assignments were excluded. The core microbiome was identified using the calc_distmx and otutab_core algorithms in USEARCH v.11, with core taxa defined as zOTUs present in more than 90% of station samples. A total of 389 fungal zOTUs were used to construct a maximum likelihood phylogenetic tree. Representative sequences of core taxa were aligned using MAFFT (29) and subsequently trimmed using trimAL (30), both with default settings. ModelFinder software was employed to determine the best-fitting phylogenetic model (31), and tree construction was performed using IQ-TREE2 software with 5,000 ultrafast bootstrap replicates. Custom annotation files were generated using the R package itol.toolkit (32) and visualized using Interactive Tree of Life (33).

### Fungal diversity and statistical analyses

Fungal diversity and statistical analyses were performed using R v.4.1 (R Core Team), including packages such as EasyAmplicon (34) and vegan (35). To ensure consistency across samples, fungal communities were normalized to a minimum sequencing depth of 40,907 reads per sample. Diversity was assessed using species richness and Shannon indices. To evaluate significant differences between seasons and station types, one-way analysis of variance followed by Tukey’s *post hoc* tests was employed. The distribution of fungal composition across seasons and station types was visualized using principal coordinate analysis (35). Analysis of similarities and multivariate permutational analysis of variance (PERMANOVA) were utilized to determine the statistical significance of compositional differences between different categories.

Relationships between fungal communities and environmental variables (e.g., such as temperature, humidity, and particulate matter (PM) levels) (Table S1) were examined using distance-based redundancy analysis, facilitated by the vegan package. Linear discriminant analysis (LDA) effect size was applied to identify significantly different fungal taxa with high abundance, referred to as biomarkers, with the effect sizes estimated using LDA scores, considering LDA > 3 and *P* < 0.05 (36). Additionally, Spearman correlation coefficient analysis was conducted in R v.4.1 (R Core Team) to explore the relationship between environmental characteristics and fungal communities within subway stations.

### Fungal trails and construction of a human pathogen database

To analyze the functional groups and ecological niches of fungal communities in subway stations, we used FUNGuild (https://github.com/UMNFuN/FUNGuild) for functional categorizations (37). To ensure the accuracy of our interpretations, we excluded the “possible” confidence level and retained only the “highly likely” and “likely” levels for fungal functional groups. Fungal communities that could not be identified or were identified as having multiple complex nutritional modes were categorized as “undefined” (38). In addition, we constructed an in-house human-associated pathogen database based on the full length of the fungal ITS region, according to the Catalog of Pathogenic Microorganisms for Human Infections formulated by the National Health Commission of China. This database contains verified representative rRNA sequences of 128 human pathogenic fungi. For the identification of human pathogens, the representative sequences of fungal zOTUs were compared with the human pathogen database using the “sintax” and “closed_ref” commands in USEARCH v.11, with a sintax_cutoff of 99% sequence similarity. Taxonomic information of all matched zOTUs was then calculated using the “sintax_summary” command in USEARCH v.11, as described previously (39, 40).

## RESULTS

### Seasonal dynamics of fungal composition in Beijing subway stations

A comprehensive analysis generated 47,483,628 high-quality sequencing reads, which were clustered into 28,390 zOTUs. The rarefaction curves of the microbial communities reached a plateau, indicating that the sequencing depth was sufficient for the samples (Fig. S1). These fungal zOTUs were classified into 3 phyla, 16 classes, 51 orders, 136 families, and 270 genera. Ascomycota is the dominant fungal phylum with 73.5% of the community, followed by Basidiomycota with 19.1%, Mucoromycota with 0.2%, and unidentified with 7.2% (Fig. S2; Table S2). Notably, the relative abundance of Basidiomycota increased from spring to winter, whereas Ascomycota decreased from spring to summer and autumn (Fig. S2). Seasonal variation was also evident at the family level, with Nectriaceae dominating in spring, Pleosporaceae in summer, and Aspergillaceae, Cladosporiaceae, and Debaryomycetaceae in autumn and winter (Fig. 1b). At the generic level, *Fusarium* and *Alternaria* were dominant in spring and summer, while *Aspergillus*, *Chaetomium*, *Cladosporium*, and *Meyerozyma* were dominant in autumn and winter (Fig. S3). Principal coordinate analysis based on Manhattan distances revealed four distinct clusters corresponding to different seasons. These clusters accounted for 25.09% of the total observed variation (Fig. 1c). PERMANOVA analysis further confirmed significant differences in fungal communities between seasons (*P* < 0.001) and station types (*P* < 0.001, Table S3). Fungal richness varied significantly across seasons. The lowest diversity was observed in summer, followed by spring, winter, and autumn (Fig. 1d; Table S3). These findings underscore the substantial impact of seasonal variations on the composition and diversity of subway fungal communities.

### Factors affecting fungal communities of subway stations

**Fig 2:**
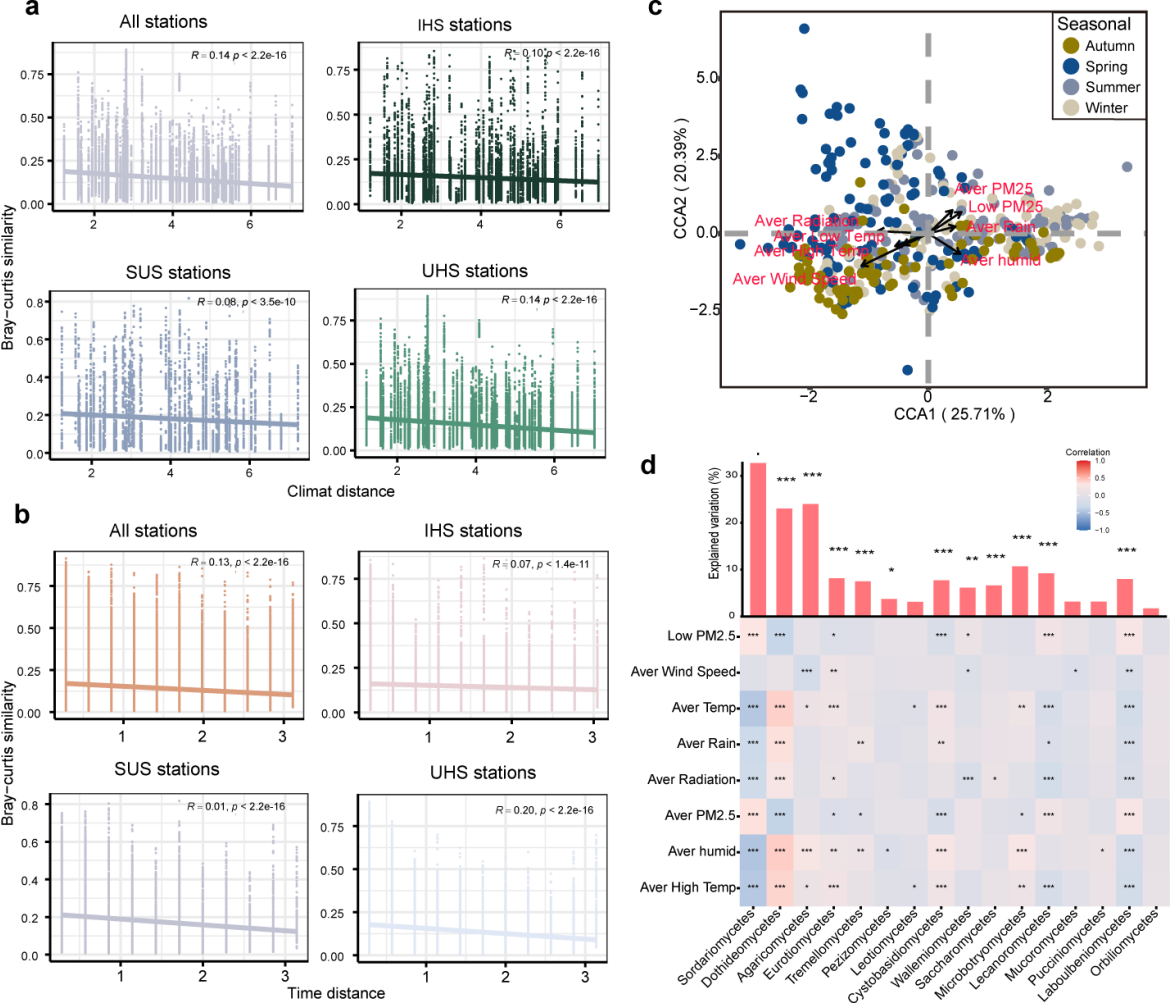
(**a**) Climate-decay relationships of fungal communities at the zOTU level in Beijing subway stations. The climate distance was calculated based on the Bray-Curtis distance between pairs of stations using the standardized climate variables. (**b**) Time-decay relationships of fungal communities at the zOTU level in Beijing subway stations. Time distance was calculated based on the Bray-Curtis distance between pairs of stations using the standardized time variables. The strengths of distance decay were measured using Spearman correlations, and the rate of decay was calculated based on the slopes of generalized linear models. (**c**) Redundancy analysis to assess the relationships between fungal communities and environmental factors. (**d**) Contributions of environmental variables to the dissimilarity of fungal phylum based on correlation analysis and best multiple regression model. The bar plots represent the percentage of explained variations of each taxon. The colors represent Spearman correlations with pink representing positive correlation and blue representing negative correlation. *P < 0.05 named significant, **P < 0.01 named very significant, ***P < 0.001 named extremely significant.

To investigate the influence of environmental factors on fungal communities, we collected climate and air pollution data for each month of the sampling period. Our analysis of distance decay patterns along both environmental and temporal gradients revealed significant (*P* < 0.001) trends across all station types (Fig. 2a and b). Further analysis using canonical correspondence analysis showed that the composition of microbial communities in stations was significantly influenced by environmental variables. Also, the Mantel test showed that the average temperature (*r* = 0.078, *P* = 0.001), average PM2.5 levels (*r* = 0.172, *P* = 0.001), average rainfall (*r* = 0.141, *P* = 0.001), average humidity (*r* = 0.234, *P* = 0.001), average wind speed (*r* = 0.030, *P* = 0.001), and average radiation (*r* = 0.1127, *P* = 0.001) played significant roles (Fig. 2d; Table S4). There appeared to be a contrasting pattern between autumn and spring (Fig. 2c). These results underline the significant impact of climatic conditions and air pollutants, such as PM2.5, on fungal communities within subway stations. Our study further identified mean PM2.5 content, mean temperature, mean rainfall, mean humidity, wind speed, and solar radiation as the main environmental factors significantly affecting fungal taxa (Fig. 2d).

### Core fungal taxa of Beijing subway stations and their seasonal dynamics

**Fig 3:**
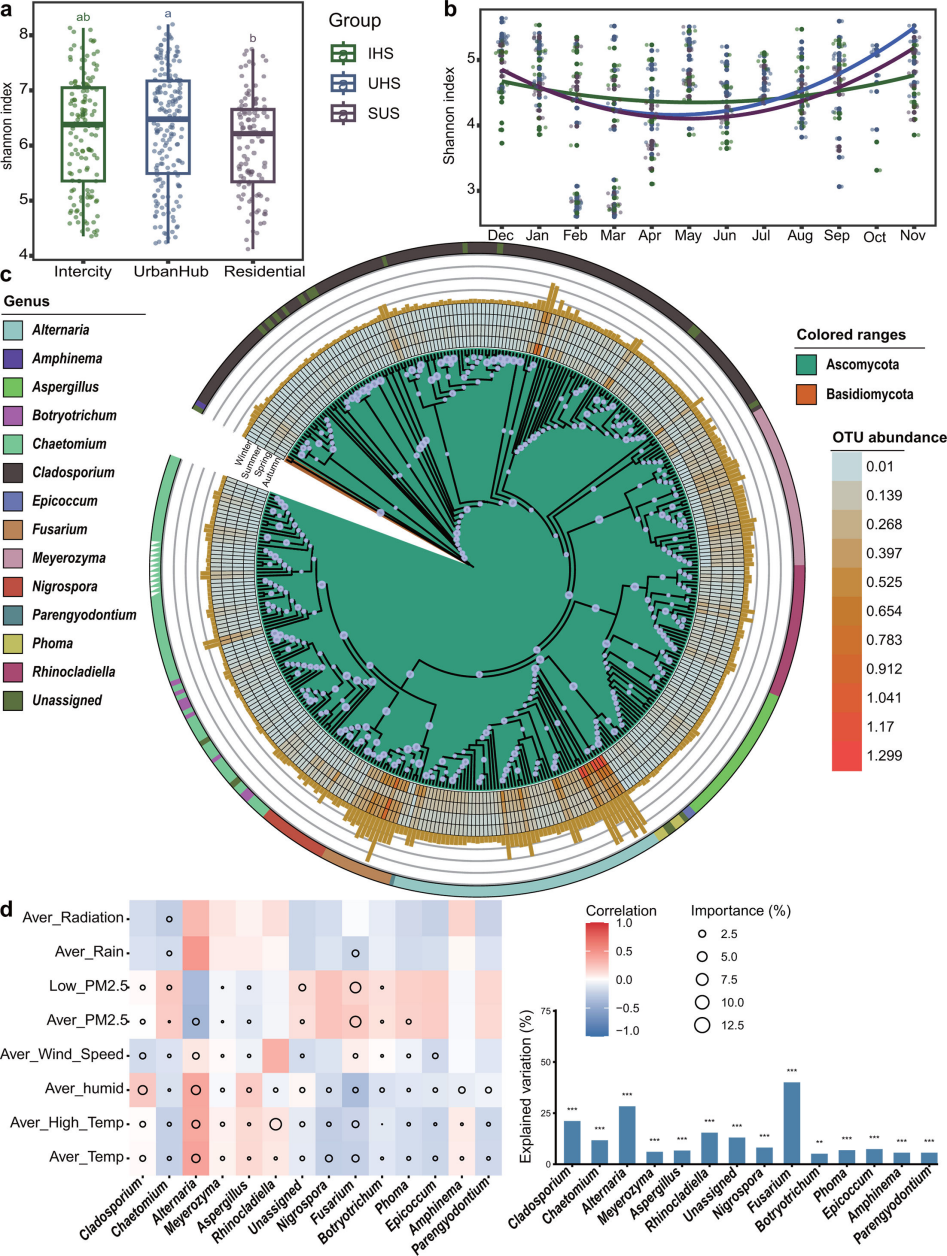
(**a**) The Shannon index of total fungal communities in different station types represented by the bar plot. The different lowercase letters indicate a significant difference between groups calculated by analysis of variance with Tukey’s honestly significant difference test. Different letters indicate significant differences at *P* < 0.05. (**b**) The dynamic change curve of 12 months in the whole year for the core fungal communities. IHS are marked in green; SUSs are marked in purple; and UHSs are marked in blue. (**c**) The phylogenetic relationships among the 389 core fungal taxa from Beijing subway stations. The bar plots at the outer layer of the tree represent the relative abundance of the core zOTUs. Each node of the phylogenetic tree is colored by the fungal phylum; the green shading of the tree indicates the taxa belong to the phylum Ascomycetes, and the orange-red of the tree indicates the taxa belong to the phylum Basidiomycetes. (**d**) Contributions and variation explanation (%) of environmental variables to the dissimilarity of core fungal genera based on correlation analysis and best multiple regression model. The colors represent Spearman correlations, with red representing a positive correlation and blue representing a negative correlation.

Located tens of meters underground, subway stations are relatively enclosed and harbor distinct core fungal communities. These core taxa remain the most important fungi in subway stations, regardless of seasonal changes and geographical locations. In our study, we identified 389 fungal zOTUs as the core taxa of Beijing subway stations, present in over 90% of the total samples. Notably, these core zOTUs represent only 1.37% of the total zOTUs but account for 41% of the total reads. The core microbial diversity varied significantly between the different subway station types, with IHS and UHS showing significantly higher core fungal diversity compared to SUS (Fig. S4). This pattern was also reflected in the total fungal communities, where IHS and UHS showed significantly higher fungal diversity than SUS (Fig. 3a). Seasonal variations were observed in the core fungal community of the different subway stations, with higher diversity in autumn and winter, lower diversity in spring and summer, and the lowest diversity in summer (Fig. 3b). To further understand these relationships, we constructed a phylogenetic tree of the core fungi based on the 389 core taxa (Fig. 3c; Table S5). At the generic level, approximately 13 different fungal genera were identified as core taxa, with *Alternaria*, *Cladosporium*, *Chaetomium*, *Nigrospora*, and *Meyerozyma* being the most abundant in Beijing subway stations. Enrichment analysis revealed specific taxonomic enrichments in different station types: Debaryomycetaceae and Didymellaceae were significantly enriched in IHS, while Aspergillaceae and Herpotrichiellaceae were significantly enriched in UHS. As for the SUS station, we found the fungal class of Dothideomycetes and unassigned fungal taxa were defined as significantly enriched in SUS. However, at the fungal family level, no specific taxa were enriched in SUS, indicating a lower level of station-specific core taxa in SUS (Fig. S5). Among all tested environmental factors, PM2.5 was identified as a key factor, correlating with higher species diversity during months with elevated levels. The study also found that *Alternaria*, *Fusarium*, and *Cladosporium*, common plant pathogenic fungi, were the most affected by environmental factors. In contrast, fungi such as *Amphinema*, *Aspergillus*, *Botryotrichum*, *Chaetomium*, *Parengyodontium*, *Meyerozyma*, and *Nigrospora* were less affected by these environmental and climatic factors (Fig. 3d). We also found humidity had greater impact in *Alternaria* and *Cladosporium* compared with others, while PM2.5 had greater impact in *Alternaria* and *Fusarium* compared with others (Fig. 3d).

### Fungal trails and community dynamics in different seasons and stations

**Fig 4:**
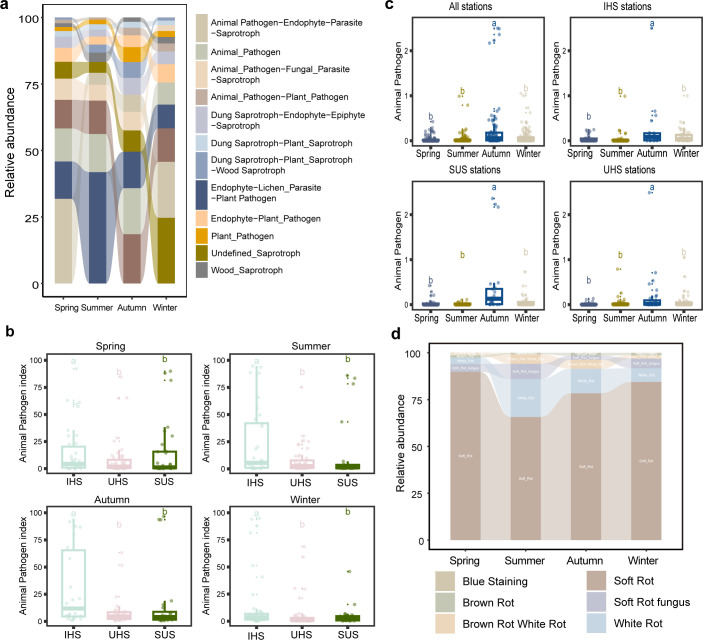
(**a**) Relative abundances of the most abundant ecotypes associated with Beijing subway stations of different seasons. (**b**) These bar plots represent the relative abundances of animal pathogens associated with different station types. (**c**) These bar plots represent the relative abundances of animal pathogens associated with Beijing subway stations of different seasons. The different lowercase letters (b and c) denote significant differences between groups calculated by analysis of variance with Tukey honestly significant difference test. Different letters indicate significant differences at *P* < 0.05. (**d**) The relative abundances of different rot-associated fungal communities in different seasons.

The FUNGuild analysis was employed to predict the potential ecological roles of the fungal communities within the subway network. The primary functional categories identified include “animal pathogen/endophyte/parasite/saprotroph,” “animal pathogen,” “animal pathogen/fungal parasite/saprotroph,” “animal pathogen/plant pathogen,” “dung saprotroph/endophyte/epiphyte saprotroph,” “dung saprotroph/plant saprotroph,” “dung saprotroph/plant saprotroph/wood saprotroph,” and “endophyte/lichen parasite/plant pathogen” (Fig. 4a). A notable variation in the functional fungal communities was observed between samples from different station types. In particular, IHS stations exhibited higher relative abundances of animal and plant pathogens compared to UHS and SUS stations in all seasons (Fig. 4b). Conversely, SUS and UHS stations showed a higher presence of plant saprotrophs and endophytes (Fig. S6 and S7), suggesting that stations with higher passenger numbers tend to harbor more medically important pathogenic fungi, while SUS stations support a greater diversity of saprotrophic and white rot fungi. Seasonal variations in pathogen-related functions were also evident. Autumn recorded the highest abundance of animal pathogen/endophyte/plant pathogen, followed by winter, spring, and summer (Fig. 4c). These findings indicate that winter is associated with a greater presence of animal and plant saprotroph-related fungal communities, whereas summer and autumn are more conductive to white rot-related fungal communities (Fig. S8 and S9).

### Human pathogen communities across different seasons and stations

**Fig 5:**
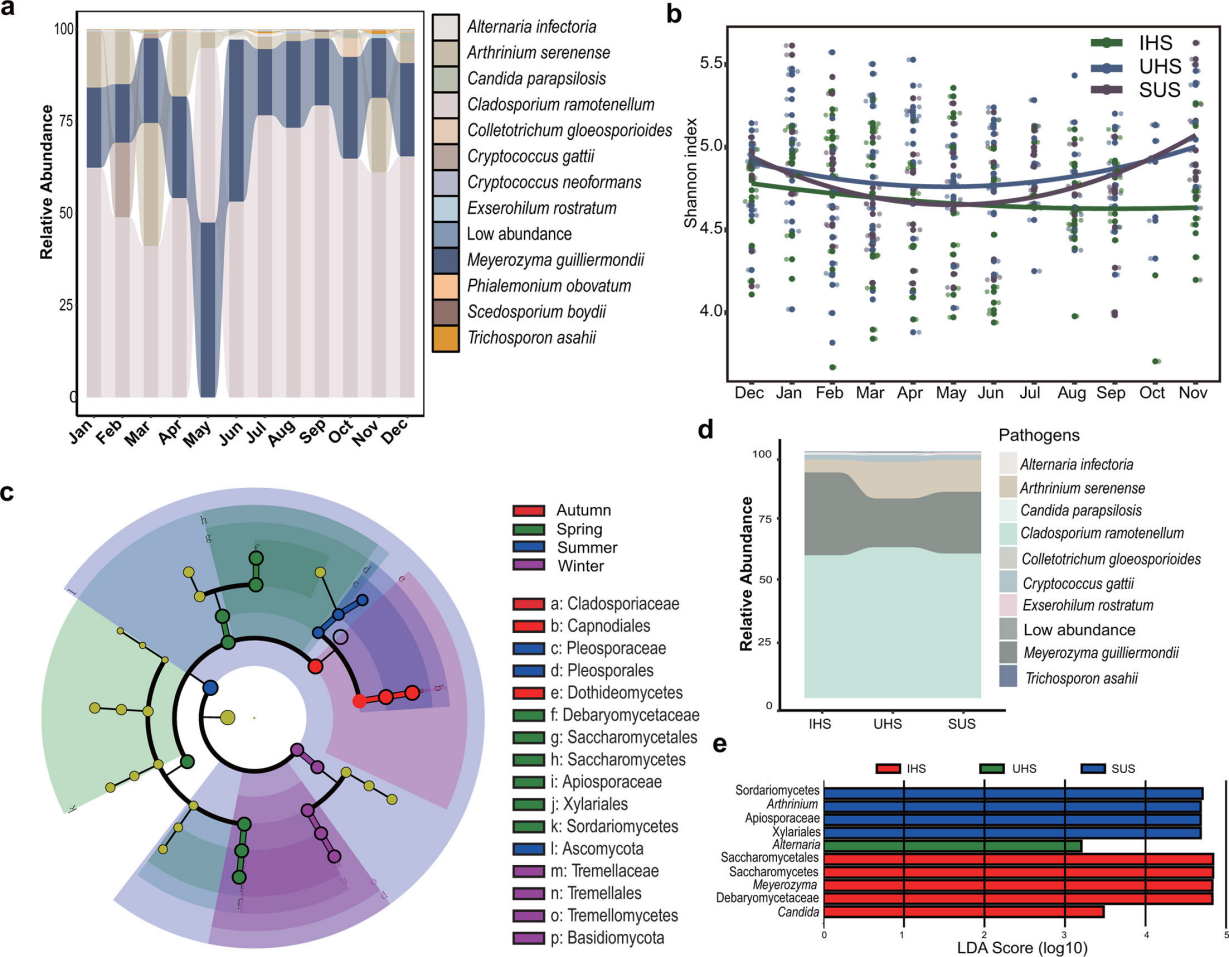
(**a**) Relative abundances of the most abundant human fungal pathogens. (**b**) The dynamic change curve of 12 months in the whole year for the human fungal pathogens. IHS are marked by green; SUS are marked by purple; and UHS are marked by blue. (**c**) A linear discriminant analysis (LDA) effect size (LEfSe) taxonomic cladogram comparing the best-discriminated fungal pathogens representing different seasons. (**d**) The relative abundances of different pathogenic fungi in three station types (**e**) LEfSe taxonomic cladogram comparing the best-discriminated fungal pathogens representing different station types. Significantly discriminant taxon nodes are colored according to the highest-ranked group for that taxon.

The detection of human fungal pathogens within subway systems has significant implications for public health, as microbial exposure to these fungi could have health concerns for people with respiratory illnesses. However, there was no “human pathogen” ecotype in FUNGuild. To facilitate this investigation, we constructed a database using the Catalog of Pathogenic Microorganisms for Human Infections, as formulated by the National Health Commission. This effort resulted in the assignment of 791 pathogen-related fungal sequences (Table S6) into 12 potentially pathogenic species according to their taxonomic classification (Fig. 5a). Several well-known human pathogens such as *Candida parapsilosis*, *Cryptococcus gattii*, *Cryptococcus neoformans*, *Trichosporon asahii*, and *Meyerozyma guilliermondii*, as well as some opportunistic pathogens such as *Exserohilum rostratum* and *Scedosporium*, were identified (Fig. 5a). Seasonal variation in pathogen diversity was observed, with higher diversity in autumn and winter and the lowest diversity in summer. This pattern may indicate more favorable conditions for transmission in autumn and winter (Fig. 5b). Enrichment analysis also revealed distinct seasonal patterns: Apiosporaceae and Debaryomycetaceae were dominant in spring, Pleosporaceae in summer, Cladosporiaceae in autumn, and Tremellaceae in winter (Fig. 5c). Across all three station types, the fungal species *Arthrinium serenense*, *Cladosporium ramotenellum*, and *Meyerozyma guilliermondii* emerged as the most dominant potential pathogens (Fig. 5d). Additionally, subway stations exhibited unique fungal profiles: IHS was enriched in the *Candida* and *Meyerozyma* groups, while UHS showed higher abundances in the *Arthrinium* and Apiosporaceae groups. However, only the *Alternaria* group was enriched in SUS, suggesting a lower presence of distinctive pathogenic fungi in these locations (Fig. 5e).

## DISCUSSION

### Composition and seasonal dynamics of mycobiome

Previous studies on subway microbiomes, which mainly focused on bacteria, have been conducted in cities such as Hong Kong, New York, and Boston (10, 12, 15). Here, we performed year-round monitoring of the air mycobiome in the world’s largest subway system. Our findings suggest a remarkably high diversity of fungal species in these subway stations. In addition to the common Ascomycota and Basidiomycota, we also found a low relative abundance of Mucoromycota. A study in South Korea revealed that Mucoromycota was found only in summer (41). However, we detected this phylum every month with two peaks in December (4.2%) and July (5.4%). Several species in this phylum could cause mucormycosis, which is a serious infection in many parts of the human body (42), in particular among the eye infections in coronavirus disease 2019 (COVID-19) patients in India (43). Therefore, the occurrence of Mucoromycota in subway air microbiome should deserve attention in public health concern because people with weakened immune systems and diabetes may be susceptible to the infection. In addition, we observed significant seasonal variations in the composition and community structure of mycobiome in the Beijing subway system. Although the seasonal dynamics have been reported in bacterial communities in other subway systems, the patterns vary. The environmental factors such as humidity are closely related to the seasonal dynamics, which can affect the fungal community of both outdoor and indoor environments. Consistent with a previous study, average humidity has a significant influence on the diversity and composition of fungal communities (44). The fungal community in the Beijing subway exhibited the highest diversity during the autumn months (September–November) and the lowest in the summer months (June–August). In contrast, bacterial diversity in the Norway subway was greater in the spring (April) and summer (June), as reported by Gohli et al. (9). These varying seasonal patterns may indicate the different microbial populations’ adaptation to local temperatures and environments.

### Impacts of stations and PM levels on the fungal communities

Previous studies have highlighted differences in fungal community compositions in subways during rush and non-rush hours (14). Extending this, our study assessed the fungal microbiome across a broader spectrum of passenger flows, selecting densely populated stations (e.g., train and airport transfer stations), as well as sparsely populated non-transfer stations in remote residential areas. The highest diversity of fungal communities and potential fungal pathogens was found in densely populated stations, suggesting that the density of subway passengers and the complexity of sources significantly influence the regional airborne fungal composition. Several studies have suggested that passenger flow may contribute to increased PM levels and the diversity of microbial composition in subway environments (45). In the Seoul subway, stations with higher passenger flow had higher mean PM concentrations (46).

Our findings highlighted the significant correlation between PM levels and fungal composition in subway stations. Higher pathogenic fungal diversity was observed in months with elevated PM levels, and lower diversity was reported in months with reduced PM levels. Also, PM level was correlated to the relative abundance of several families and fungal composition. At the genus level, the abundance of medically important representatives such as *Alternaria*, *Aspergillus*, and *Candida* was positively correlated with PM2.5 and PM_10_ during August to October in Bangkok subway (14). A similar finding was reported in another study, showing a positive correlation between PM levels and the occurrence of human respiratory diseases or allergies, such as those caused by *Aspergillus*, *Cladosporium*, and *Penicillium* (47, 48). Earlier studies suggest many opportunistic fungal pathogens were significantly differently distributed among various air qualities. Hence, the PM level in the subway system could pose a direct health risk not only through chemical pollutants but also through particulate matter, which contains fungal spores of pathogens that cause severe illness and lung and respiratory infections (49, 50). For instance, in northern China, air pollution and PM levels increase during the autumn and winter seasons, which is consistent with epidemiological data showing an increase in respiratory and allergic illnesses during this period (51). As the study was performed during the COVID-19 pandemic, the implementation of strict infection control measures in the subway system could influence the composition and structure of fungal communities. For example, people choosing work-from-home and flexible working hours could reduce the daily passengers in the subway station, leading to reduced community diversity. Extensive and proactive disinfection and sanitization procedures may also influence the compositions and structure of fungal diversity.

### Health implication of core mycobiome

The relatively confined space and the nature of being tens of meters underground may create a unique environmental reservoir for microbial colonization and adaptation. This unique environment contributes to the development of distinct fungal core taxa. A previous study by Loos et al. (49) demonstrated that fungal communities are more environmentally specific than bacterial communities, as evidenced by the analysis of 1,580 samples from various biomes. They found the specialists’ core taxa account for a small fraction of the total microbial diversity in each kingdom (with <1.3% of fungal genera) while contributing greatly to the microbial community compositions (49). Similar to previous research, our results support this finding by showing that although core zOTUs represent only 1.4% of the total OTUs, they account for 41.0% of the total sequence reads. This highlights the high abundance and potentially critical role of these core fungal taxa in the fungal community. In our study, we found that 12 genera dominated the core fungal taxa in the Beijing subway system. Most of them are ubiquitous fungi, such as *Alternaria*, *Aspergillus*, *Cladosporium*, and *Parengyodontium*. While *Aspergillus* spp. are well-recognized causes of fungal lung disease, non-*Aspergillus* fungi such as *Cryptococcus* and *Pneumocystis* also can play a significant role in pulmonary infections (52). Indeed, several of these pathogens can be discovered in the transit system in our investigation. Most core taxa are commonly found not only in other subway systems (14, 41) but also in soils, dominating different ecosystems and geographies (53, 54). Their ability to spread by wind and their flexible trophic capabilities may have contributed to their global distribution (49, 55, 56). A previous global metagenomic map of urban microbiomes revealed 31 species in the core microbiome, with only one high-confidence eukaryote identified, *Saccharomyces cerevisiae*. However, in our study, *Saccharomyces* had a low abundance, especially during the winter months, while another yeast genus, *Meyerozyma*, dominated. *Meyerozyma*, delineated from *Pichia*, is frequently reported from various environments, human skin and mucosa, and one species, *M. guilliermondii*, is a rare yeast pathogen that can cause invasive infections in hospitalized malignant patients (57). Another core genus, *Rhinocladiella*, deserves much attention because *Rhinocladiella* spp. have emerged as opportunistic serious pathogens in both clinical and environmental contexts. Transmission of these subway core pathogenic fungi in passenger traffic may exacerbate the risk of disease outbreaks such as invasive fungal infections, allergic fungal infections, and asthma (18, 58).

### Spatiotemporal dynamics of functional composition

Given the important role of domestic animal diseases in human infections (59), we monitored the relative abundance of animal pathogens across seasons and station types. The annotation revealed by the FUNGuild database indicated that the fungal composition showed distinct seasonal successional patterns, particularly the human and animal pathogens. The results show that these factors, such as average PM2.5 and average temperature, have greatly influenced the composition of potential human pathogenic fungi, with the highest diversity in autumn. This trend is consistent with air pollution and respiratory disease patterns of different seasons in Beijing (60). The abundance of animal and human pathogenic fungi also varied between station types. IHS stations exhibited the highest diversity, followed by UHS stations, while SUS stations had the lowest diversity, possibly due to passenger flow (41). There was a greater association of *Candida* and *Meyerozyma*, which are human commensals, in IHS, compared to *Xylaria* in SUS. The IHS group includes four railway stations and a major transfer station to the airport, bringing together a large influx of travelers from across the country and the world. This influx may facilitate the convergence and dissemination of pathogenic fungi carried by passengers (61). Similarly, the UHS group, consisting of five transfer stations connecting two to three subway lines, serves as a key hub in the Beijing subway network and plays a potentially important role in the dissemination of pathogenic fungi (62). Our analysis revealed distinct fungal community patterns in SUS stations, characterized by two key observations: an elevated presence of white rot-associated fungi (including *Bjerkandera* spp., *Perenniporia japonica*, *Rigidoporus* spp., and *Trametes hirsuta*) and a reduced diversity of human pathogens, alongside a greater abundance of plant pathogens. The lower prevalence of human pathogens aligns with previous research by Li et al. (63), who documented significantly decreased relative abundance of human-associated pathogens in forested areas compared to urban and residential environments. The higher occurrence of both white rot fungi and plant pathogens can be attributed to the geographical context of SUS stations, which are predominantly situated in rural areas with dense vegetation.

### Conclusion

This study represents the first comprehensive investigation into the composition and dynamics of mycobiome within the world’s largest subway system. Our findings revealed a rich diversity of the subway mycobiome that varies seasonally and is influenced by environmental factors, particularly PM2.5 levels. We detected potential clinically important fungi, including well-known pathogens such as *Candida* spp. and *Cryptococcus* spp., which peak in autumn and winter, suggesting favorable conditions for proliferation in these seasons. Station types had a significant influence on the mycobiome, as the highest diversity of pathogenic fungi was discovered in IHS and UHS. The identified core taxa, which comprise cosmopolitan fungi and pathogenic fungi, provide critical insights and important information for understanding the transmission of fungi in urban environments. This study reveals the complex interplay between environmental factors and fungal communities in the Beijing subway, highlighting the potential public health risks of human pathogenic fungi in the subway environment.

## Data Availability

Scripts and pipelines for subway bioinformatic analysis and plotting used in this study are available at https://github.com/XinJason/SubwayMycobiome.git. The raw paired-end Illumina sequence data have been deposited to Genome Sequence Read Archive in the National Genomics Data Center, China National Center for Bioinformation, under BioProject accession number PRJCA028767, and all the data can be accessed through Genome Sequence Archive number CRA020909.

## SUPPLEMENTAL MATERIAL

Fig. S1 spectrum.01626-25-s0001.pdf The rarefaction curves of the microbial communities from air samples in each station.

Fig. S2 spectrum.01626-25-s0002.pdf Relative abundances of the most abundant fungal phyla linked to Beijing subway stations.

Fig. S3 spectrum.01626-25-s0003.pdf Relative abundances of the most abundant fungal genera linked to Beijing subway stations.

Fig. S4 spectrum.01626-25-s0004.pdf Shannon index of mycobiome from different types of stations.

Fig. S5 spectrum.01626-25-s0005.pdf LDA effect size (LEfSe) taxonomic cladogram comparing the best-discriminated fungal pathogens representing different station types.

Fig. S6 spectrum.01626-25-s0006.pdf The seasonal dynamics of relative abundances of plant saprotrophs associated with different station types

Fig. S7 spectrum.01626-25-s0007.pdf The relative abundances of endophytes associated with different station types.

Fig. S8 spectrum.01626-25-s0008.pdf The seasonal dynamics of relative abundances of white rot fungi in different station types.

Fig. S9 spectrum.01626-25-s0009.pdf The seasonal dynamics of relative abundances of plant saprotrophic fungi in different station types.

Supplemental tables spectrum.01626-25-s0010.xlsx Tables S1 to S6.

ASM does not own the copyrights to Supplemental Material that may be linked to, or accessed through, an article. The authors have granted ASM a non-exclusive, world-wide license to publish the Supplemental Material files. Please contact the corresponding author directly for reuse.
