# Supplementary figures and images for: Dynamic airborne mycobiome in the metropolitan city transit system is driven by seasonality and station type
Source: Microbiol Spectr. 2025 Sep 25;13(11):e01626-25. doi: 10.1128/spectrum.01626-25 (PMC12584724; doi:10.1128/spectrum.01626-25)

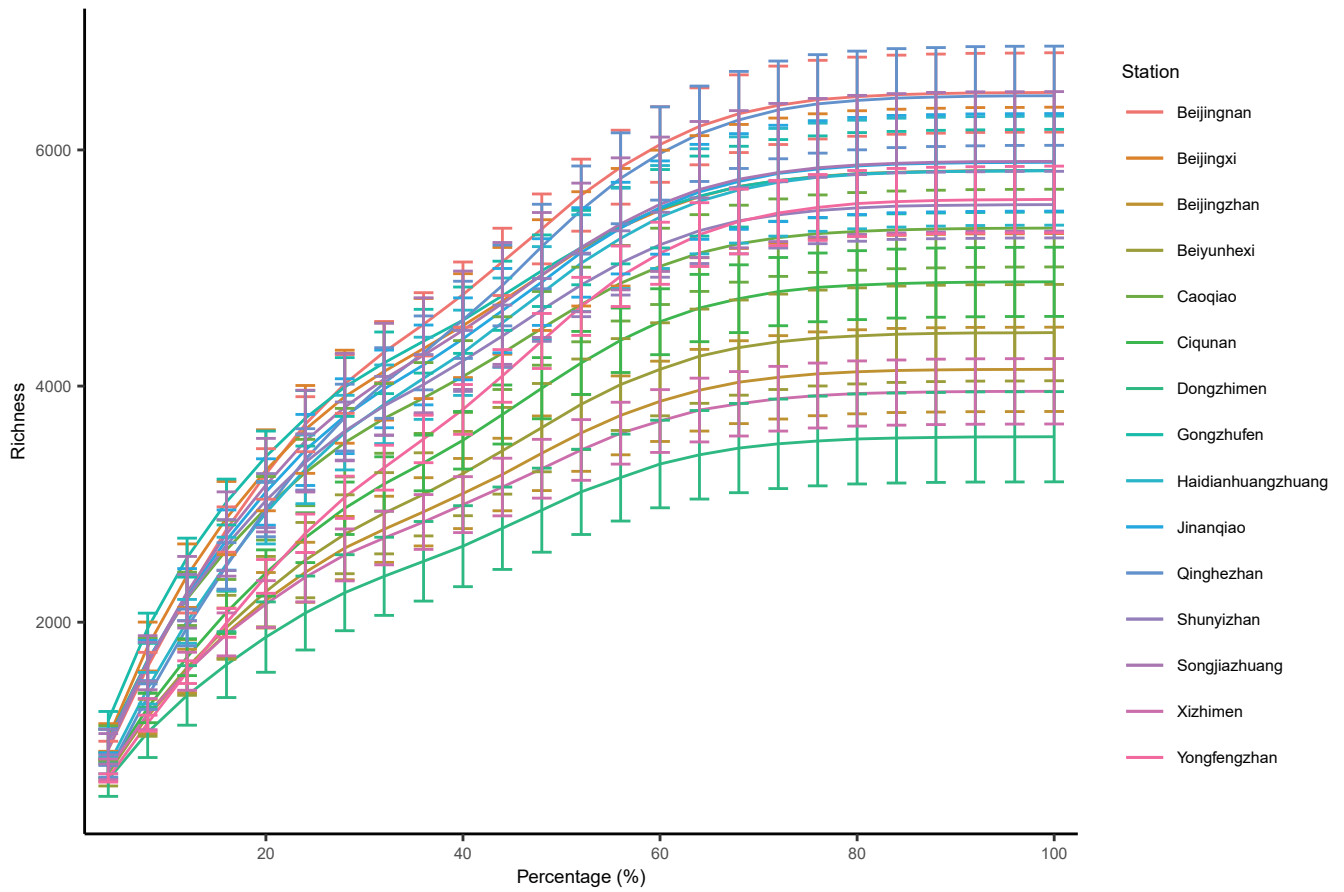

Fig. S1 The rarefaction curves of the microbial communities from air samples in each station

Supplement: Fig. S1 — The rarefaction curves of the microbial communities from air samples in each station. [file spectrum.01626-25-s0001.pdf]

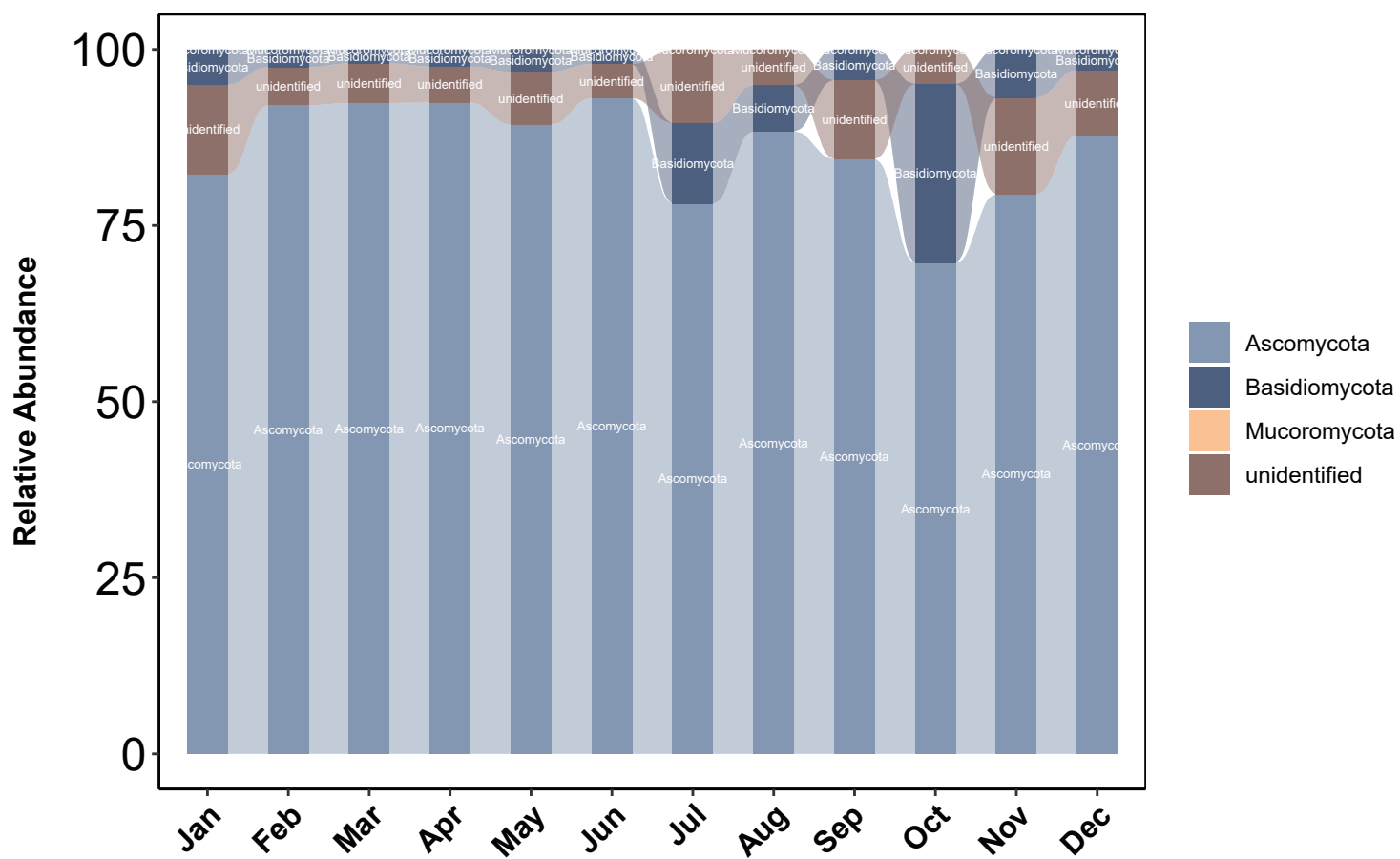

Fig. S2 Relative abundances of the most abundant fungal phyla linked to Beijing subway stations

Supplement: Fig. S2 — Relative abundances of the most abundant fungal phyla linked to Beijing subway stations. [file spectrum.01626-25-s0002.pdf]

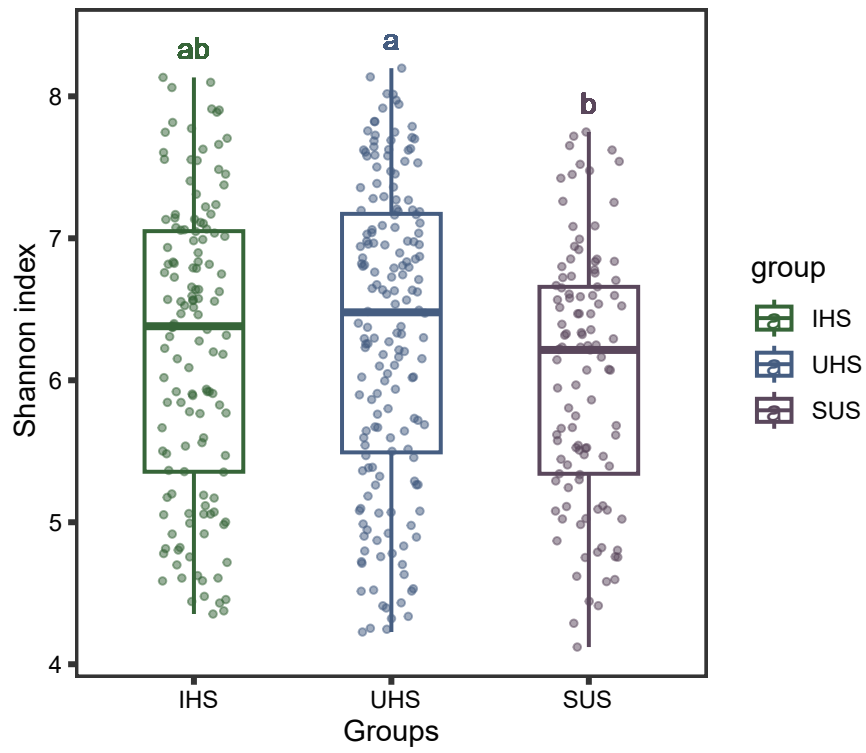

Fig. S4 Shannon index of mycobionite from different types of stations.

Supplement: Fig. S4 — Shannon index of mycobiome from different types of stations. [file spectrum.01626-25-s0004.pdf]

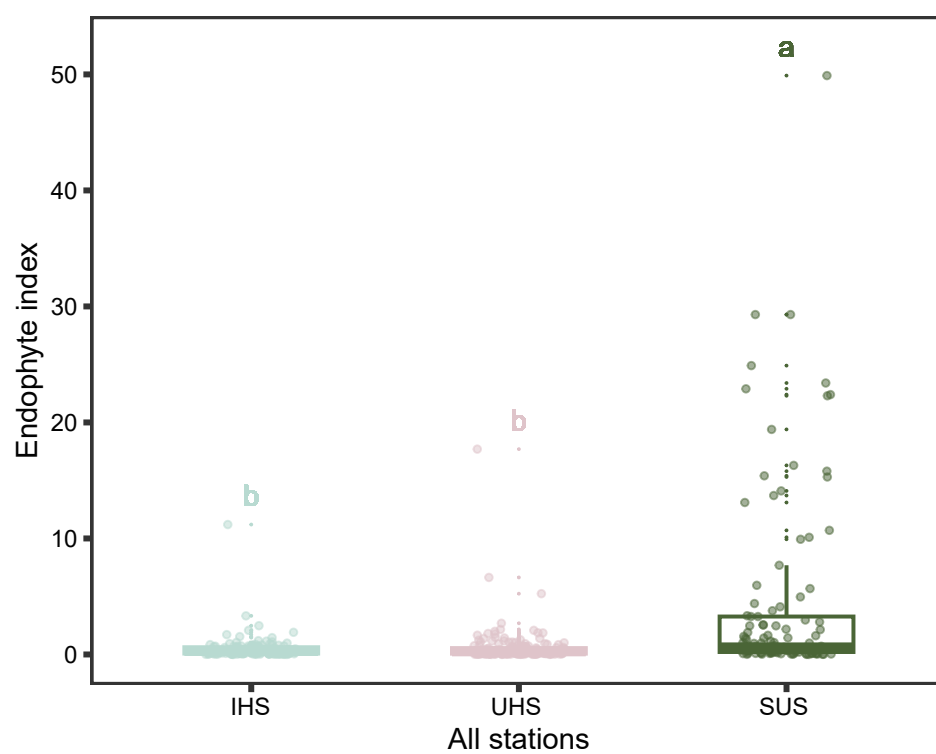

Fig. S7 The relative abundances of endophyte associated with different station types.

Supplement: Fig. S7 — The relative abundances of endophytes associated with different station types. [file spectrum.01626-25-s0007.pdf]
