# Supplementary material for: Dynamic airborne mycobiome in the metropolitan city transit system is driven by seasonality and station type
Source: Microbiol Spectr. 2025 Sep 25;13(11):e01626-25. doi: 10.1128/spectrum.01626-25 (PMC12584724; doi:10.1128/spectrum.01626-25)
Supplement: Fig. S5 — LDA effect size (LEfSe) taxonomic cladogram comparing the best-discriminated fungal pathogens representing different station types. [file spectrum.01626-25-s0005.pdf]

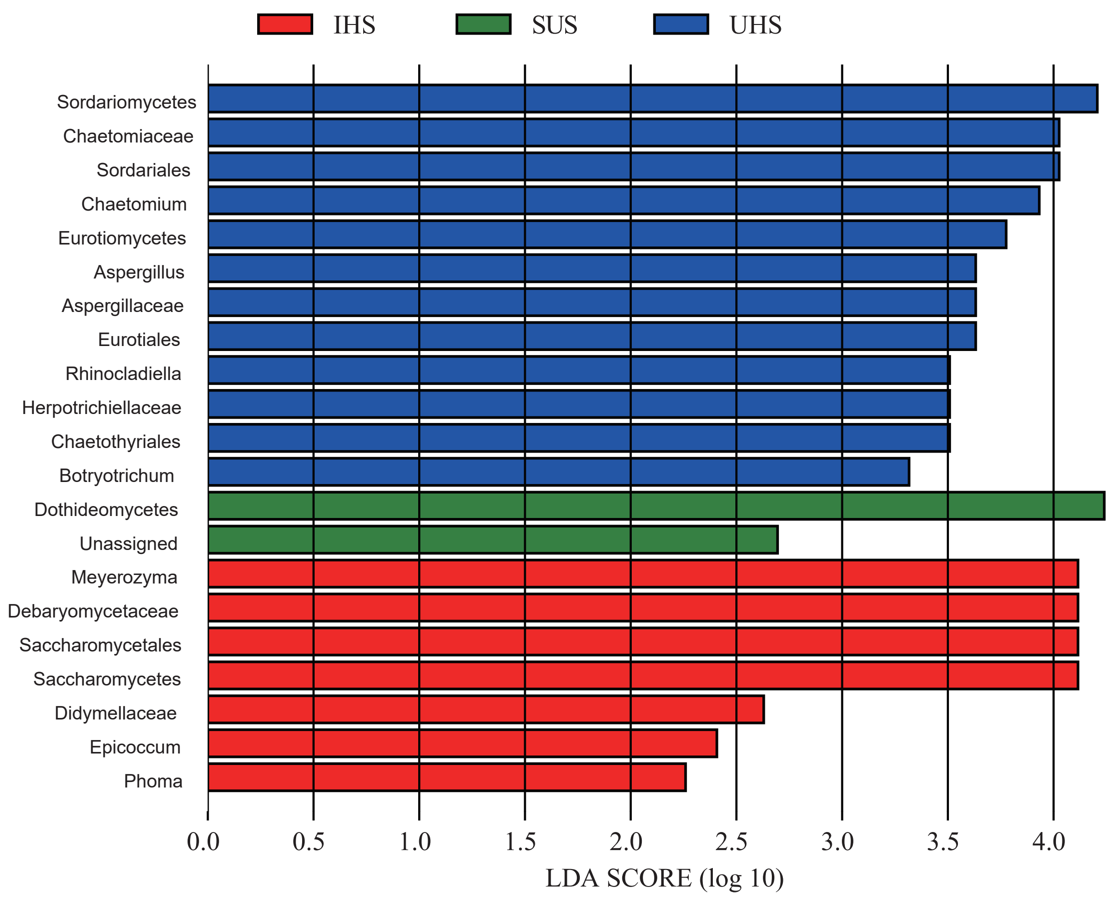

Fig. S5 LDA effect size (LEfSe) taxonomic cladogram comparing the best-discriminated fungal pathogens representing different station types.
