# Supplementary material for: Dynamic airborne mycobiome in the metropolitan city transit system is driven by seasonality and station type
Source: Microbiol Spectr. 2025 Sep 25;13(11):e01626-25. doi: 10.1128/spectrum.01626-25 (PMC12584724; doi:10.1128/spectrum.01626-25)
Supplement: Fig. S8 — The seasonal dynamics of relative abundances of white rot fungi in different station types. [file spectrum.01626-25-s0008.pdf]

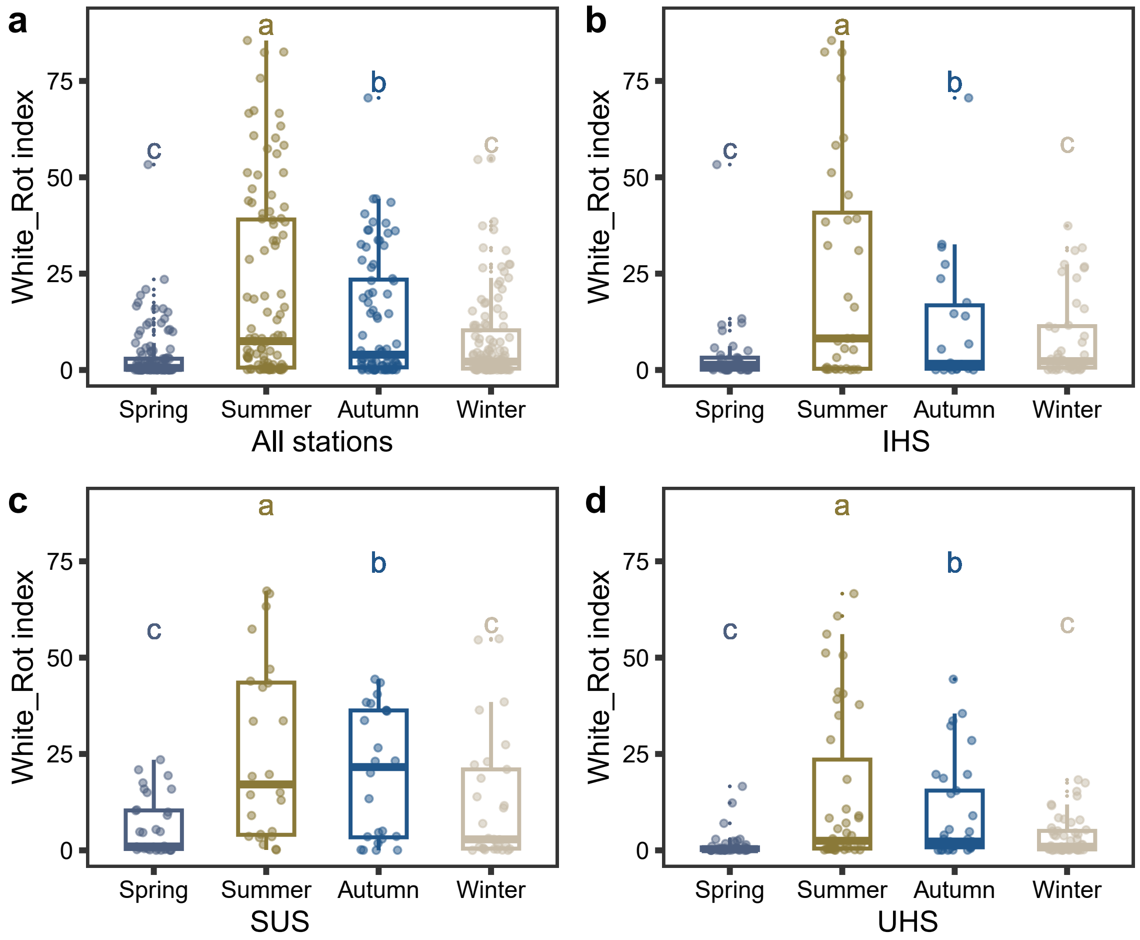

Fig. S8 The seasonal dynamics of relative abundances of white rot fungi in different station types.
